# The ethics of donation and transplantation: are definitions of death being distorted for organ transplantation?

**DOI:** 10.1186/1747-5341-2-28

**Published:** 2007-11-25

**Authors:** Ari R Joffe

**Affiliations:** 1 Stollery Children's Hospital, 3A3.07 8440 112 Street, Edmonton, Alberta, Canada T6G 2B7

## Abstract

A recent commentary defends 1) the concept of 'brain arrest' to explain what brain death is, and 2) the concept that death occurs at 2–5 minutes after absent circulation. I suggest that both these claims are flawed. Brain arrest is said to threaten life, and lead to death by causing a secondary respiratory then cardiac arrest. It is further claimed that ventilation only interrupts this way that brain arrest leads to death. These statements imply that brain arrest is not death itself. Brain death is a devastating state that leads to death when intensive care, which replaces some of the brain's vital functions such as breathing, is withdrawn and circulation stops resulting in irreversible loss of integration of the organism. Circulatory death is said to occur at 2–5 minutes after absent circulation because, in the context of DCD, the intent is to not attempt reversal of the absent circulation. No defense of this weak construal of irreversible loss of circulation is given. This means that paents in identical physiologic states are dead (in the DCD context) or alive (in the resuscitation context); the current state of death (at 2–5 minutes) is contingent on a future event (whether there will be resuscitation) suggesting backward causation; and the commonly used meaning of irreversible as 'not capable of being reversed' is abandoned. The literature supporting the claim that autoresuscitation does not occur in the context of no cardiopulmonary resuscitation is shown to be very limited. Several cases of autoresuscitation are summarized, suggesting that the claim that these cases are not applicable to the current debate may be premature. I suggest that brain dead and DCD donors are not dead; whether organs can be harvested before death from these patients whose *prognosis *is death should be debated urgently.

## Introduction

In the commentary "Clarifying the paradigm for the ethics of donation and transplantation: was 'dead' really so clear before organ donation," Shemie argues in favor of the concept of brain death (BD) being death itself, and defends the rationale for accepting the DCD donor as dead [1]. On more detailed examination, I suggest that his arguments are flawed for several reasons. This commentary aims to discuss the limitations of Shemie's arguments, and identifies some of the persistent dilemmas that have been overlooked. Specifically, I suggest that the brain arrest argument shows that BD is *not *death, that intensive care *does *replace some critical functions of the brain, that the DCD patient is *not *in the irreversible state called death, that death has always been understood as 'not capable of being reversed', and that minimizing the information on auto-resuscitation is ill-advised. This has enormous implications for clinical practice involving organ donation and the diagnosis of death.

## Discussion

### The brain arrest argument shows that BD is not death (but, can *lead *to death)

Shemie suggests that "BD is better understood as 'brain arrest' [BA], characterized by the complete and irreversible loss of clinical brain function [1]." For Shemie, and in this commentary, BD and BA can be used interchangeably. Shemie writes (all italics added): "Breathing replacement machines merely interrupt the way brain failure *leads to *cardiac arrest [1]." Shemie further writes that "Advances in organ support and replacement technologies teach us about the mechanics of death. Survival of...the human organism is related to adequacy of oxygenated blood flow...There are 3 basic mechanisms [leading to death]: a) primary cardiac arrest *leading to *arrest of the circulation b) primary respiratory arrest, which via loss of oxygen *causes a secondary *cardiac arrest, or c) primary BA, which via interruption of respiratory control *causes a secondary *respiratory then cardiac arrest. Regardless of initial disease state, all critical illnesses *threaten life *in this way [1]." He further states that life sustaining technologies are deployed to interrupt this sequence and therefore "to reverse the underlying *life threatening *state...the removal of those applied *life sustaining *technologies must occur for 'natural' death and cardiac arrest to ensue [1]." Thus, by these descriptions in defense of BD, we can conclude that 'BA' *leads to death *when it is allowed to result in irreversible loss of circulation. If 'BA' threatens life, and leads to death, it follows that BA cannot be death itself. What BA must be is a *mechanism leading to *death. This is compatible with what Shemie writes in another paper: the "basic physiological *mechanism *of death [in BA occurs]...via interruption of airway control and respiratory drive cause a secondary respiratory arrest and then cardiac arrest... [BA] threatens life in this manner [2]." Shemie also implies this when he writes, when referring to cardiac arrest, that "the event may be cardiac arrest, but *death only occurs if it leads to *an accompanying [permanent] loss of circulation [1]."

To see that this is true, we should consider that the reasoning behind BA being a "better way" to understand BD can be applied to other situations. For example, if we label kidney failure as "kidney arrest", we could write that "dialysis to perform the function of the kidneys merely interrupts the way kidney arrest leads to death;" but, this does not mean we are misled to consider 'kidney arrest' death. Whether the state is named 'BD', or 'BA' (or 'kidney arrest') does not provide a rationale for why this state *is *death. Shemie's statements clearly show that BA is not death; it will only *lead to *death if we stop the ventilator and allow a *secondary *respiratory and then a cardiac arrest to be followed by irreversible loss of circulation.

### Misunderstanding of brain death (intensive care replaces some critical brain functions)

Shemie writes: "ICU care does not replace *any *functions of the brain...any degree of brain failure, including BD, can be sustained indefinitely with mechanical ventilation and vigilant care [1]." This is a problematic statement requiring some background information. The standard and accepted philosophical concept (or definition) of death is the irreversible loss of the integration of the organism as a whole [3,4]. In other words, when the organism is no longer integrated as a whole, there is an irreversible progression to increasing entropy, and this state of dis-integration *is *death. There are said to be two medical/biological criteria for this state of death: brain death, and circulatory death [3]. When the brain is 'dead', some have argued, there is irreversible loss of the integration of the organism as a whole, and *therefore *this inevitably results in irreversible circulatory arrest in a very short time, and this is why BD *is *death. The bedside tests (often called 'the criteria' for brain death) are used to determine that the brain is dead; that is, that there is irreversible loss of the function of the entire brain, including the brainstem.

If death is the loss of integrative unity of the organism, then the 'BA' patient "sustained indefinitely" by intensive care must not be dead. If the 'BA' patient (organism) can be "sustained indefinitely" by intensive care, as has been reported many times in the literature, then he/she must have integrative unity, and he/she must have had brainstem functions (such as breathing) taken over by intensive care [5,6]. If breathing is a function of the brain, accounting for the need for an apnea test in confirming BD, then the ventilator must have taken over this function of the brain. If temperature regulation and electrolyte balance is a function of the brain, then the BD patient having external warming for temperature control, and electrolyte balance maintained by desmopressin must have had these functions of the brain replaced. This is similar to an anuric renal failure patient having dialysis to replace the function of the kidneys, or a diabetic patient having insulin injections to replace the function of the pancreas, both of which "merely interrupt" the way these diseases lead to cardiac arrest.

It is tempting to consider the brain dead patient to have 'integrative unity' that is different from the patient with kidney failure or diabetes receiving artificial support. However, as noted by several authors, this suggestion is surprisingly hard to defend when one looks at the details [6-12]. Kamm writes that "some argument would be needed for why artificiality in cause matters when 'lower' integrated functioning of the organism results but not when 'higher' integrated functioning results...It is not clear why completely replacing the component at the apex should be different from replacing a component somewhere else in the loop [13]." For example, a brain dead patient can be compared to a patient with an "intact" brain, and also to a patient with a brain only capable of initiating infrequent ineffective breaths when the PaC02 reaches 61 mmHg. These patients are both considered alive, because they have integrative unity. However, the latter patient has no more integrative unity than a brain dead patient. The breathing is just an attempt to respire, not even enough to sustain the organism; it is not integration, just an attempt at it [13]. The integration of the organism is present in this case independent of brain activity, just as it is in brain death.

If 'BA' is death, there must be some concept of death other than integrative unity that it satisfies; however, what this concept is has never been clarified. One candidate concept of death may be the irreversible loss of the capacity for consciousness. Shemie suggests as much when arguing that "it takes less than 20 seconds for *cortical *brain function to stop after cardiac arrest...any permanent absence of brain blood flow beyond 20 seconds will lead to permanent absence of *brain *function [1]." If it is true that death is the irreversible loss of the capacity for consciousness, then we should be willing to consider the patient in an irreversible vegetative state at "the point in time after which consequences occur." This means we should bury or cremate or autopsy or harvest organs from patients in an irreversible vegetative state, while breathing and moving but without consciousness [7]. Clearly, and in all countries, this concept of death fails, and we are left with the persistent problem of somehow finding a concept of death that can allow BD to be a criterion for death.

### Avoiding the issue of the irreversibility of death of the organism (focusing on the cell)

Shemie makes some statements about cellular death that at first seem relevant to the discussion about death, but on reflection are not. He discusses that "the complete and irreversible cessation of all cell life has become increasingly indefinable," and "that at the cellular level, 'irreversible cessation of the entire brain' is elusive [1]." He later writes "The so-called time of death has always been an arbitrary moment within an overlapping segment of decreasing vital functions and increasing quantity of cell death. No matter how convenient it is to assume that death and life are opposite and that a patient is either dead or alive, the process of death is a gradual event where organs and cells die at different rates, depending on their resistance to the lack of oxygen. As a result, the biology of death cannot be a moment... [1]." These statements about cellular death are generally interesting, but are devoid of information relevant to determining the death of the organism. The problem is that these variable times of death of each cell are not directly related to the *concept *of death of *the organism*. The time of death is that moment when the organism is dead and hence no longer alive; conceptually, it is the moment when there is irreversible loss of integrative unity of the organism. The variable times of death of each cell do not mean that death of the organism is "an arbitrary moment."

We are being misled to believe that when defining a moment of death of the organism the requirement is that all the cells of that organism have undergone necrosis. Shemie implies that this is what critiques of DCD and BD require as the "line of 'unequivocal death'." I do not believe that this has been required by the literature describing concerns about DCD or BD. What has been required is that the organism that is dead is in an irreversible state of no circulation such that the organism has lost integrative unity. Pointing out that at the cellular level not all cells have undergone necrosis when death has occurred is not contributing to the discussion about death at the organism level. This interesting information detracts from the real issue: when has irreversible loss of integration of the organism occurred? At present, we know that this has occurred when there is an irreversible loss of circulation. There may be other criteria for death; however, what these are, and how to justify them is unknown. If BD or DCD patients are dead, they must satisfy some concept of death other than the "irreversible loss of the integration of the organism as a whole;" however, this new concept of death has not been described to date.

### The issue of the irreversibility of death (irreversible usually means 'not capable of being reversed')

Shemie suggests that death as a biological event "is relative to the context [in which it occurs]." For example, "the ability to restore the circulation depends on the location of the [cardiac] arrest, a predetermined ethical decision regarding level of medical intervention, the types of interventions available..., and the types of interventions actually used [1]." By this, he means "the issue is not whether the body or brain circulation and function can be resumed (because it can), but rather, whether it will be [1]" (i.e. the present state of death is contingent on a predicted future event, suggesting backward causation [14]). What Shemie does not do is defend this point of view. He has simply implied that he accepts this weak construal of irreversible loss of circulation. This means that purported *permanent *cessation of circulation is equivalent to the *irreversible *state of death. Why we should accept this weak construal of irreversible is nowhere discussed or defended, other than stating that "DCD in particular, has by necessity enhanced the rigour of the determination of death [1];" this, by clarifying that we mean *permanent *loss of circulation which occurs at 2 minutes in Pittsburgh, 5 minutes in Canada, 10 minutes in some places in the United States and United Kingdom, and 20 minutes in Sweden [15-18]. One can argue that this clarification was developed solely for organ donation (utilitarian) purposes.

The ethical concern of *when *to declare a person dead, with *irreversible *cessation of circulation, is central to the debate regarding DCD. Many authors have argued for a weak construal of irreversibility, whereby the state *will not *be reversed (i.e. there is a "do not resuscitate" order and cardiac auto-resuscitation is not possible) [15,16]. This construal is weaker than generally stated because it is actually based on the premise that loss of circulation *ought not *be reversed, rather than *will not *be reversed. Others argue for a stronger construal, whereby the state *cannot *be reversed even if resuscitation is attempted [14,19,20]. With resuscitation attempted, absent circulation for over 10 minutes can be reversed and not result in death or BD. By the weak construal of irreversibility, patients in the identical physiological state *are *dead or alive based on their location and prediction of a future event (attempted resuscitation). What needs to be decided is if it is sensible that one patient whose heart has stopped for 2 minutes (in Pittsburgh) or 5 minutes (in Canada) is pronounced dead for organ donation, while another identical patient whose heart has stopped for 5 minutes and then has CPR is not pronounced dead and survives. The commonly held meaning of irreversible is 'not capable of being reversed;' this means that after 10 or 15 minutes of absent circulation, without the intention to intervene, the patient's *prognosis *is death, and their *physiological state *is dying [14]. It seems that the prognosis of death has been confused (or conflated) with the diagnosis of death. Admitting that one can reverse the process of death is an admission that the individual is not dead. Is a drowning man dead because no one will swim out to save him? Or is he merely going to die?

### Was dead really so clear before organ donation? (the irreversibility of death was)

Shemie writes that "the so-called time of death...is and always has been a line within an overlapping segment of decreasing cell functions and increasing cell death... [1]." The title of Shemie's commentary asks "Was 'dead' really so clear before organ donation [1]?" I suggest that the notion of death *was *clear before organ donation.

Shemie writes that in diagnosing death in the past "Observation and confirmation was not required and the irreversibility of death was not a practical concern, *although diagnostic errors were made *[1]." Acknowledging that "diagnostic errors were made" shows that what death is has been clear in the past. Outside the context of organ donation, when one was claimed to be dead based on the irreversible loss of circulation, if the patient was subsequently revived (by auto-resuscitation or by intervention) and clearly alive, one simply had to admit that the pronouncement of death was *incorrect*. I do not believe that in this situation one would continue to insist that the diagnosis of death was correct, and that somehow the patient was revived from the irreversible state known as death. This shows that death in the past was understood as an irreversible state of dis-integration of the organism. However, in the context of DCD, we are now forced to "enhance the rigour of the determination of death," and would in this situation of revival have to explain somehow that the patient in the irreversible (or "permanent") state 'death' has now somehow become alive. I do not know how proponents of DCD would explain this.

### Auto-resuscitation (it cannot be dismissed so easily)

Two claims are made regarding resuscitation. First, Shemie claims that "the limits of brain resuscitation are commonly quoted as 4–10 minutes [1]." While this may be common teaching, it is inaccurate. The literature shows that in animals and humans, absent circulation for over 10–15 minutes can be followed by resuscitation without BD. This is not just survival of individual cells, it is survival of the organism as a whole without the state of BD occurring. For example, Hossmann et al report a cat who survived with integrative neurological function after one hour of global cerebro-circulatory arrest at normothermia [21], and Kolata reported that 80% of 10 rabbits with brain ischemia for 20 minutes and 75% of 4 rabbits with brain ischemia for 30 minutes survived [22]. It is interesting that these animals were given heparin after the ischemia, something done for DCD donors that may prevent injury to the brain. A study in Spain of combined in and out of hospital arrests showed the following mortality at hospital discharge with <4 min, 4–10 min, 10–20 min, and >20 min delay to start of CPR: 56.5%, 61.1%, 81%, and 100% [23].

The second claim is that "no autoresuscitation after withdrawal of life sustaining treatment has been described beyond 2 minutes in the absence of CPR suggesting that the provision of CPR is a confounding condition [1]." This claim merits careful scrutiny. There are many cases of autoresuscitation described in the literature, and some have indeed been hampered by inadequate monitoring [24]. Some cases have had constant EKG monitoring with constant observation; 6 of these had autoresuscitation at 5–8 minutes after absent circulation and asystole (implying also absent brain blood flow) (Table 1) [25-30]. Some cases have had arterial line monitoring in addition to continuous EKG and observation, with 3 of these having autoresuscitation at 3–5 minutes after absent circulation and asystole (Table 1) [31-33]. Some cases with inadequate monitoring but with constant observation were found to have autoresuscitated 8–10 minutes after absent circulation with asystole (Table 1) [34-36]. It has been hypothesized that these cases may be due to reversal of auto-PEEP once ventilation is stopped, or delayed delivery of resuscitation drugs to the heart. These hypotheses seem inadequate to account for the time delay of several minutes before autoresuscitation has occurred [37]. Auto-PEEP resolves within seconds of stopping ventilation, as shown by studies documenting lung derecruitment within seconds of disconnection from a ventilator [38]. Drug delivery to the heart during asystole is difficult to explain. Attributing all the autoresuscitation cases to CPR may be ill-advised considering that there have been no prospective studies.

**Table 1:** Selected reported cases of autoresuscitation.

**Author**	**Age (yr)**	**Diagnosis**	**Rhythm**	Min*	**Outcome**	**EKG**	**AL**	Obs**
Letellier [25]	80	Pulmonary edema	asystole	5	normal	+	-	+
Voekkel [26]	55	Sudden death	asystole	7	death at 3 d	+	-	+
MacGillivray [27]	76	COPD	asystole	5	death 24 hr later	+	-	+
Rosengarten [28]	36	asthma	EMD	5	normal	+	-	+
Abdullah [29]	93	sepsis	asystole	5	not stated	+	?	+
Al-Ansari [30]	63	COPD	asystole	3	normal	+	-	+
Frolich [31]	67	MI	asystole	5	normal d3; death d7	+	+	+
Casielles-Garcia [32]	94	hemorrhage	EMD	3	death at 18 d	+	+	+
Maleck [33]	80	sepsis	asystole	<5	death at d2	+	+	+
Quick [34]	70	hyperkalemia	asystole	8	normal	-	-	+
Ben-David [35]	66	sudden VF	asystole	10	normal	-	-	+
Monticelli et al [36]	78	MI	asystole	>10	death at 19 hr	-	-	+

AL: arterial line; COPD: chronic obstructive pulmonary disease; EKG: electrocardiogram; EMD: electromechanical dissociation; MI: myocardial infarction; VF: ventricular fibrillation. *Min: time in minutes from stopping resuscitation in the stated rhythm to return of circulation. **Obs: refers to continuous clinical bedside observation.

Shemie claims that autoresuscitation after withdrawal of life sustaining treatment in the absence of CPR has never been described [1]. This is based on data from studies that will be scrutinized here [see Additional file 1] [39-43]. This data is limited by: poorly described patient selection criteria, no description of whether there was continuous clinical monitoring other than EKG, no arterial line monitoring, many patients who did have resuscitation attempts, and *only 5 cases where EKG monitoring was stated to have continued more than 2 minutes after loss of cardiac activity *[39-43]. A more recent report of 12 patients who had withdrawal of ventilation after catastrophic brain injury showed that 2 "showed a salvo of 5 to 20 heartbeats 1.23 and 6 minutes after asystole, followed by EKG silence. An arterial catheter in two of these patients did not record measurable tracings during the cardiac activity. Four recordings showed broad undefined complexes after 5, 7, 9, and 10 minutes after initial cardiac arrest... [44]" A report from Pittsburgh on 15 DCD patients stated that "after 2 mins monitor activity was not recorded, so continuously recorded data after this time are not available [45]." This information suggests that the data is not adequate to state that autoresuscitation is "extremely rare and is likely negligible [1]."

### Laying my cards on the table

I have argued that brain death is not death itself. It is a devastating neurological state with a dismal prognosis, but not death. I agree with Shemie that it leads to death when (and only when) ventilation is stopped, and therefore breathing stops, followed by cardiac arrest, followed by irreversible loss of circulation. This results in an irreversible state of loss of integration of the organism, and this is death.

Further, I believe that at 2 to 10 minutes after loss of circulation the DCD donor is not dead. This is because there is not necessarily irreversible loss of circulation (i.e. loss of circulation could still be reversed) and hence irreversible loss of integration of the organism. When exactly this state of irreversibility occurs is an important question. At present this is not known; however, it is known that it is not at even 10 minutes after cardiac arrest.

Whether I am challenging the practice of organ donation is another question. The question is not whether organ donors are dead (because they are not). The question is whether organs can be harvested before death from patients whose *prognosis *is death, and hence be a contributing cause of death. My argument is that this is the current practice, and this is also precisely what needs to be debated urgently. Is organ harvesting before death violating respect for persons and using them as a means?

## Conclusion

This commentary suggests that many of the arguments in support of BD and DCD may be questioned. If BD threatens life, and leads to death, it must not be death itself. If intensive care only interrupts the way BD leads to death, then intensive care must take over some of the brain functions, allowing the organism to be sustained indefinitely in a state of integrative unity. If the DCD donor is dead after 2–5 minutes of cardiac arrest, then patients in identical physiologic states actually *are *dead or alive depending on the context, the state of death is contingent on a future event (whether resuscitation is attempted), and the commonly used meaning of irreversible as 'not capable of being reversed' is abandoned. The literature supporting the claim that autoresuscitation does not occur in the context of no resuscitation attempts is more limited than usually acknowledged. Several cases of autoresuscitation in the literature were well monitored and described, suggesting that ignoring these cases may not be warranted. These problems have major implications for the practice of medicine today. I suggest that brain death is not death itself, and that the DCD donor is not dead even at 10 minutes after absent circulation. Whether organs can be harvested before death from patients whose *prognosis *is death should be debated urgently.

## Competing interests

The author(s) declare that they have no competing interests.

## Supplementary Material

Additional file 1 Case series documenting lack of auto-resuscitation. The table provides details of the studies quoted by several groups to justify the claim that auto-resuscitation does not occur after more than 2 minutes after withdrawal of life support. The limitations of these studies are described in the table. Click here for file
