# Supplementary material for: The ethics of donation and transplantation: are definitions of death being distorted for organ transplantation?
Source: Philos Ethics Humanit Med. 2007 Nov 25;2:28. doi: 10.1186/1747-5341-2-28 (PMC2211498; doi:10.1186/1747-5341-2-28)
Supplement: Additional file 1 — Case series documenting lack of auto-resuscitation. The table provides details of the studies quoted by several groups to justify the claim that auto-resuscitation does not occur after more than 2 minutes after withdrawal of life support. The limitations of these studies are described in the table. [file 1747-5341-2-28-S1.doc]

**Additional file 1:**

# Case series documenting lack of autoresuscitation

| Study (year) | n | Selection criteria | Monitoring >2 min* | AL | Cont obs | Author’s conclusions | Comment |
| --- | --- | --- | --- | --- | --- | --- | --- |
| Stroud et al [39] (1947) | 23 | “When practical, the electrodes were attached to the moribund patient before clinical death…tracings were taken intermittently or continuously until the string became motionless.” Age 10-87yr. | stated for n=2 | 0 | not stated | “Permanent standstill occurred without VF in 50% of cases.” | n=2 not monitored at death, leaving a true n=21. |
| Enselberg [40] (1952) | 43 | “EKGs were recorded for varying lengths of time before, during, and after death…resuscitative measures were applied in 22 cases.” Age 8-80yr. | not stated | 0 | not stated | “Recurring asystoles or ventricular standstills are common and often appear to be self-limited.” | “It is possible that in many cases the recording of terminal EKGs may have been stopped upon the appearance of a long asystole, before true cessation.” |
| Robinson [41] (1912) | 7 | “EKG records obtained from 7 patients before and during the actual stoppage of the heart…There were naturally many failures to obtain records, especially when fatalities occurred suddenly.” Age 8mo to 37yr. | stated for n=1 | 0 | not stated | “Cardiac activity continued from 6-35min after all the usual clinical signs of death had occurred.” | -Case 5 had resumption of cardiac rhythm at a rate of 33 bpm “after a stoppage of 2 ½ minutes.”  -Case 7 had no evidence of cardiac activity “17 min post mortem. Because the records were not satisfactory, a more detailed analysis is not possible.” |
| Willius [42]  (1924) | 6 | “…six patients in whom almost continuous EKG records were obtained from 10min to 7hr 32min preceding death.” Age 29-58yr (based on information for n=4). | not stated (4), several minutes (1), 1min 3.04 sec(1). | 0 | not stated | “The changes occurring in the mechanism of the human heart preceding and during death are variable…” | - |
| Rodstein et al [43] 1970 | 31 | “A series of aged individuals in whom terminal EKGs and necropsies were available…Lead II was then continuously recorded until electrical activity ceased…The time of cessation of electrical activity- electrical death- was recorded. Where clinically indicated, the usual resuscitative measures were employed.” Ages 73-101yr. | stated for n=1. | 0 | not stated | “The majority of deaths from all causes showed an EKG pattern of the dying heart…” In review of the literature they report “survival times after clinical death [of up to 50min].” | “In 7 (23%) of the 31 patients, electrical death terminated in VF…One patient terminated with a rapid VT.” |
| TOTAL | 110 | Selection criteria poorly described. | n=5 | n=0 | not stated | variable terminal EKG patterns | cannot determine if autoresuscitation occurs |

AL: arterial line; EKG: electrocardiogram; VF: ventricular fibrillation. Cont obs: continuous observation. *Monitoring >2min: refers to ongoing EKG monitoring for >2min after death was pronounced based on EKG asystole or VF.
